# Interaction between Different Pharmaceutical Excipients in Liquid Dosage Forms—Assessment of Cytotoxicity and Antimicrobial Activity

**DOI:** 10.3390/molecules23071827

**Published:** 2018-07-23

**Authors:** Dániel Nemes, Renátó Kovács, Fruzsina Nagy, Mirtill Mező, Nikolett Poczok, Zoltán Ujhelyi, Ágota Pető, Pálma Fehér, Ferenc Fenyvesi, Judit Váradi, Miklós Vecsernyés, Ildikó Bácskay

**Affiliations:** 1 Department of Pharmaceutical Technology, Faculty of Pharmacy, University of Debrecen, Debrecen 4032, Hungary; nemes.daniel@pharm.unideb.hu (D.N.); mmirtill95@gmail.com (M.M.); poczok.niki@freemail.hu (N.P.); ujhelyi.zoltan@pharm.unideb.hu (Z.U.); agota713@gmail.com (Á.P.); feher.palma@pharm.unideb.hu (P.F.); fenyvesi.ferenc@pharm.unideb.hu (F.F.); varadi.judit@pharm.unideb.hu (J.V.); vecsernyes.miklos@pharm.unideb.hu (M.V.); 2 Department of Medical Microbiology, Faculty of Medicine, University of Debrecen, Debrecen 4032, Hungary; kovacs.renato@med.unideb.hu (R.K.); nagyfru@freemail.hu (F.N.)

**Keywords:** excipient interaction, surfactant, liquid dosage forms, cytotoxicity, preservative, Caco-2 cells

## Abstract

Nowadays, the safety of parabens as pharmaceutical preservatives is debated. Recent studies investigated their interference with the oestrogen receptors, nevertheless their carcinogenic activity was also proved. That was the reason why the re-evaluation of the biocompatibility and antimicrobial activity of parabens is required using modern investigation methods. We aimed to test the cytotoxic, antifungal and antibacterial effect of parabens on Caco-2 cells, *C. albicans*, *C. parapsilosis*, *C. glabrata*, *E. coli*, *P. aeruginosa* and *S. aureus*. Two complex systems (glycerol—Polysorbate 20; ethanol—Capryol PGMC™) were formulated to study—with the MTT-assay and microdilution method, respectively—how other excipients may modify the biocompatibility and antimicrobial effect of parabens. In the case of cytotoxicity, the toxicity of these two systems was highly influenced by co-solvents and surfactants. The fungi and bacteria had significantly different resistance in the formulations and in some cases the excipients could highly modify the effectiveness of parabens both in an agonistic and in a counteractive way. These results indicate that with appropriate selection, non-preservative excipients can contribute to the antimicrobial safety of the products, thus they may decrease the required preservative concentration.

## 1. Introduction

Although tablets and capsules are the most popular types of pharmaceutical dosage forms, different oral liquid formulations (syrups, herbal extracts, suspensions, emulsions, etc.) still have specific therapeutic indications, mainly in paediatrics. Flavouring is a crucial part of these formulations because patient compliance is highly dependent on the taste of the product. Usually they contain high amount of sweet carbohydrates (glycose, fructose, maltitol, xylitol, sorbitol, etc.), which can be metabolized by different microorganisms, thus the product can be easily contaminated [1]. It must be noted, that these liquid preparations are opened and closed multiple times during their life-time and each application increases the possibility of contamination. In order to avoid it, an appropriate amount of preservatives must be used, which can kill or inhibit the growth of bacteria, fungi and other unicellular. The exact mechanism of action of preservatives is unclear in some cases, but as the cell membrane is the only common subcellular component in these microbes, they mostly distort the structure of the membrane resulting in several consequences [2]. Their cytotoxicity is mostly based on these effects as well [3].

**Figure 1:**
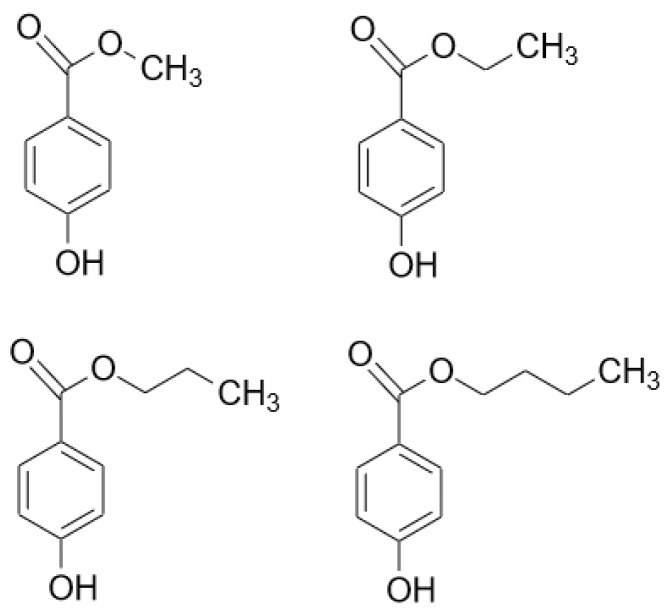
Chemical structure of the most commonly used parabens in growing alkyl chain length: methyl paraben, ethyl paraben, propyl paraben, butyl paraben.

One of the most widely used group of pharmaceutical preservatives is the parabens. They are derivatives of 4-hydroxybenzoic acid in the form of its carboxylic esters. The most commonly used parabens (Figure 1) are methyl paraben (MP) (E218), ethyl paraben (EP) (E214), propyl paraben (PP) (E216), butyl paraben (BP), heptyl paraben and their respective sodium salts. The longer the alkyl chain, the lower the solubility in water is. Hence, some co-solvent such as ethanol is usually required to increase their solubility and it must also be noted, that the sodium salts are less frequent in different formulations. Generally, they are considered as synthetic compounds, but in the recent years many natural sources were found [4,5,6]. They are preferred in the pharmaceutical and cosmetic industries, because of their odourless and tasteless characteristics, great chemical stability over a wide range of pH values and a broad spectrum of antimicrobial activity [7].

The esters of 4-hydroxybenzoic acid also have certain well-known risks. In the case of topical application, contact dermatitis is a well-known problem [8,9] however, the latest results are controversial, describing a low occurrence of allergic reactions caused by parabens [10,11] or a severe influence on sensitization [12]. Recent studies have indicated the carcinogenic effect of parabens, as they interfere with oestrogen receptors [13,14]. Furthermore, in vivo evidence suggests that urine paraben levels can be associated with menstrual cycle problems [15]. They are able to penetrate through the skin from cosmetic products [16,17]. Their direct cytotoxic behaviour has been reported on corneal epithelial cells [18], on dermal fibroblasts [19] and on liver cells [20]. Paraben exposure is not only restricted to the users of cosmetics [21], as they can pass through the placenta [22] and can be measured in the milk of lactating mothers [23]. These results suggest a decline in the use of these 4-hydroxybenzoic acid derivatives in oral and topical formulations during the next few years. 

An oral, liquid pharmaceutical preparation contains many excipients, which is the reason why cytotoxicity tests of each chemical by itself is not enough to gain a comprehensive view of the biocompatibility profile of the product. There are only few studies on how the biocompatibility of an excipient is influenced if other components are present in the test systems. However, in order to get authorized by governmental authorities, the whole product cannot be toxic, but positive interactions might decrease the appropriate concentration of additives i.e., the quantity of preservatives may also be reduced. However, serious cytotoxicity values may be measured, if the excipients can potentiate their harmful effects [24]. As the cytotoxic effects of surface-active agents are well-known [25], they might have synergetic antimicrobial activity with preservatives. Different co-solvent mixtures can have different biocompatibility profiles and might modify the toxicity of preservatives, increasing their effect on the cell membranes by creating a better chemical environment at the site of action [24].

In this study, our objective was to investigate the cytotoxicity and antimicrobial properties on Caco-2 cells and on various pathogenic microorganisms of different 4-hydroxybenzoic acid derivatives alone and in two complex co-solvent systems to explore interferences between the preservatives and the component of the co-solvent systems. Caco-2 cells are widely applied as an in vitro model of human gastrointestinal transport and mainly used as a monolayer rather than individual cells, however several assays are performed prior to reach complete integrity, such as end point or non-invasive cell viability assays (MTT assay, LDH test, RT-CES, etc.) [26]. In our antimicrobial experiments, our test solutions were tested on clinically relevant pathogens: *S. aureus* as a Gram-positive facultative anaerobe, *E. coli* and *P. aeruginosa* as a Gram-negative aerobes and *C. albicans* as the most common fungal pathogen and *C. parapsilosis* and *C. glabrata* as the top *Candida* species opportunistic pathogens different from *C. albicans* [27]. 

**Figure 2:**
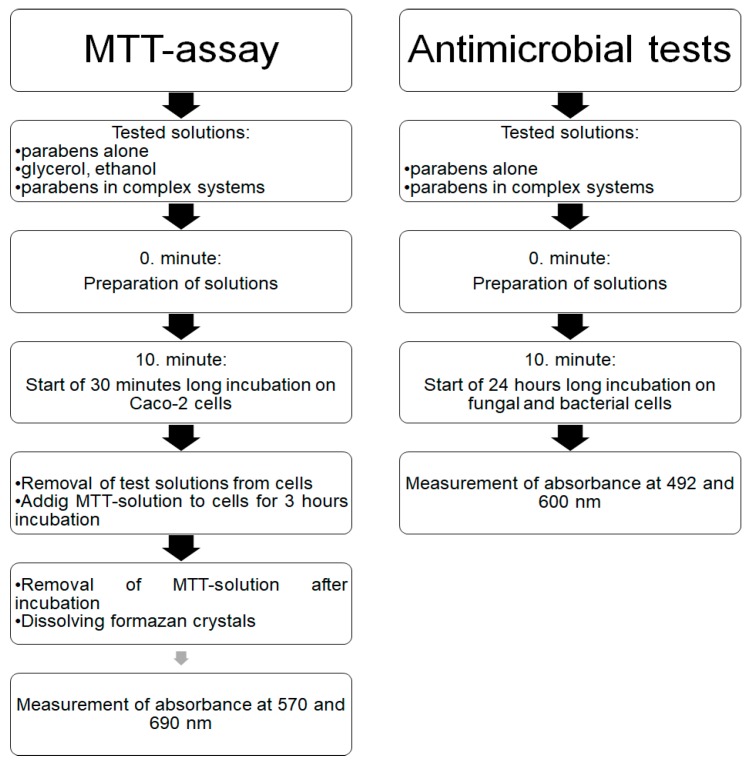
Experimental design.

The formulations of the investigated systems contain a co-solvent and a surface-active agent. The first formulation (S1) consisted of 30% (*v*/*v*) glycerol and 0.002% (*v*/*v*) Polysorbate 20. The surfactant of the second formulation (S2) was 0.5% (*v*/*v*) Capryol PGMC™ and the parabens in the form of their 10 (*w*/*w*)% solutions, dissolved in 70% (*v*/*v*) ethanol. Table 1 and Table 2 summarize the composition of every solution used in our experiments. The experimental design is presented in Figure 2.

Test solutions were prepared in situ, 10 min before the inoculation for antimicrobial investigations. Caco-2 cells were incubated for 30 min with the test solutions, then these solutions were removed and the MTT-solution was added for a 3 h long reaction. The converted formazan crystals were dissolved in appropriate solvents after the unreacted MTT was removed. Absorbance was measured at two different wavelengths and the cell viability was calculated. After seeding the bacterial and fungal cells in appropriate concentrations into 96-well microplates, a 24 h long incubation was started. Optical density was measured at two wavelengths at the end of the incubation period.

## 2. Results

### 2.1. Cytotoxicity Tests

#### 2.1.1. Cytotoxicity of Parabens

**Figure 3:**
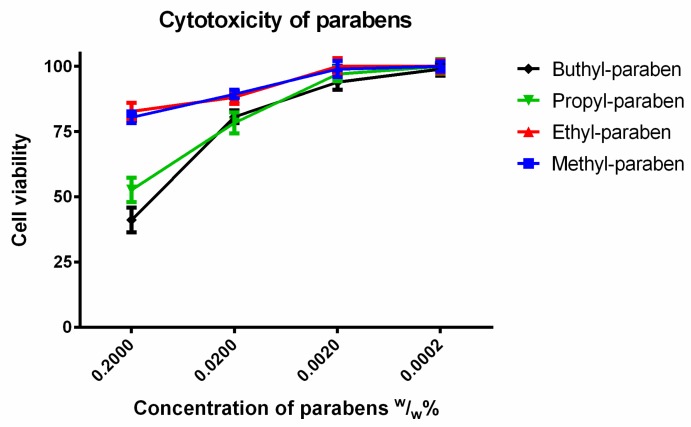
Cytotoxicity of parabens on Caco-2 cells measured by MTT-assay. Cell viability was expressed as the percentage of the absorbance of the untreated control cells. Data expressed as mean ± SEM, *n* = 12. Cell viability of the samples at 0.2000; 0.0200; 0.0020; 0.0002 (*w*/*w*)% concentrations: MP: 81% ± 2.4%; 89% ± 1.6%; 99% ± 3.1%; 100% ± 2%; EP: 83% ± 3.3%; 88% ± 2.6%; 100% ± 3.1%; 100% ± 2.5%; PP: 53% ± 4.7%; 78% ± 4%; 97% ± 2.7%; 100% ± 2.7%; BP: 41% ± 4.6%; 81% ± 2.4%; 94% ± 2.9%; 99% ± 2.5%.

In order to mimic the dilution of samples in the gastrointestinal tract, the cytotoxicity of parabens was measured in tenfold, hundredfold and thousand-fold dilutions (Figure 3). The samples were diluted by PBS. At 0.2 (*w*/*w*)% butyl and ethyl paraben had significantly higher cytotoxicity than methyl and ethyl paraben, which had similar toxicity patterns. There was a linear relationship between the cytotoxicity and the dilution ratio of different paraben derivatives. The more concentrated samples decreased the cell viability and resulted in significant cytotoxicity. The higher the ratio of dilution of parabens, the better the cell viability of the Caco-2 cell line was.

#### 2.1.2. Cytotoxicity of Solvents

**Figure 4:**
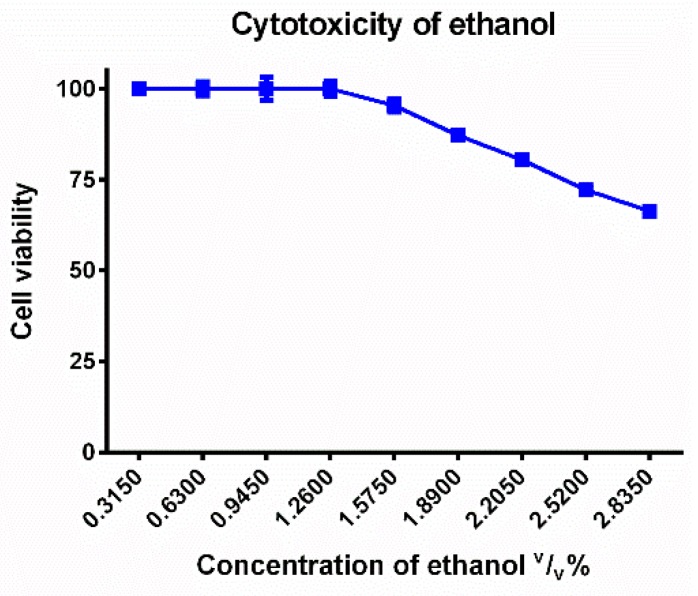
Cytotoxicity of ethanol measured by MTT-assay. Cell viability expressed as the percentage of the absorbance of the untreated control cells. Data expressed as mean ± SEM, *n* = 12. Cell viability of the samples at the different concentrations: 100% ± 0.2%; 100% ± 1.8%; 100% ± 3.1%; 100% ± 2%; 95% ± 1.7%; 87% ± 0.5%; 81% ± 1.1%; 72% ± 0.9%; 66% ± 1%.

**Figure 5:**
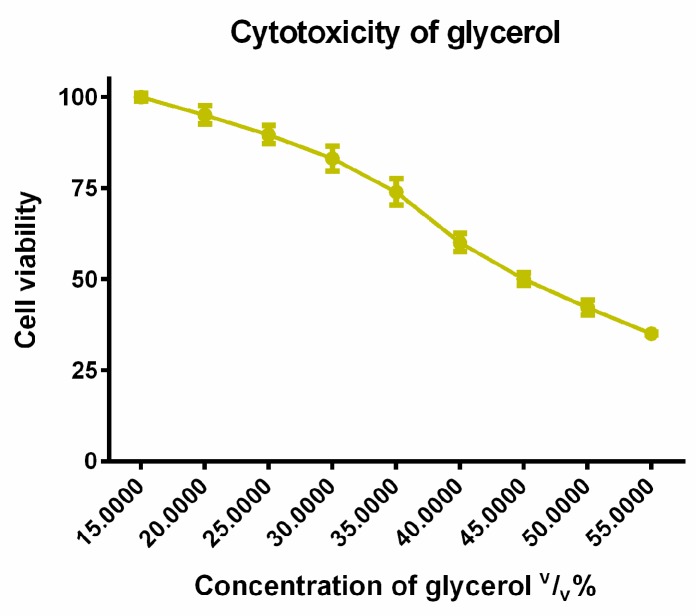
Cytotoxicity of glycerol measured by MTT-assay. Cell viability expressed as the percentage of the absorbance of the untreated control cells. Data expressed as mean ± SEM, *n* = 12. Cell viability of the samples at the different concentrations: 100% ± 1.1%; 95% ± 2.5%; 90% ± 2.5%; 83% ± 3.4%; 74% ± 3.6%; 60% ± 2.5%; 50% ± 1.7%; 42% ± 2%; 35% ± 0.4%.

Ethanol and glycerol were tested in different concentrations diluted with phosphate buffered saline (PBS) for cytotoxicity experiments. As it can be seen on Figure 4 and Figure 5, the cell viability decreased in a concentration dependent manner in the case of these solvents. The IC_50_ (the inhibitory concentration value, where the 50% cell viability was measured by an MTT test) of glycerol was 45 (*v*/*v*)%. In our complex systems, the concentrations of glycerol were 30 (*v*/*v*)%, 3 (*v*/*v*)%, 0.3 (*v*/*v*)%, 0.03 (*v*/*v*)% which were lower than this inhibitory concentration.

The concentrations of ethanol (1.75 (*v*/*v*)%, 1.4 (*v*/*v*)%, 0.14 (*v*/*v*)%, 0.014 (*v*/*v*)%) in complex systems were applied for cytotoxicity and antimicrobial tests. Based on this cytotoxicity test, the IC_50_ value cannot be determined in these concentration ranges. The cell viability slightly decreased according to the concentration, but the highest concentration (1.75 (*v*/*v*)%) decreased the cell viability significantly (80 ± 1.7%).

#### 2.1.3. Cytotoxicity of Formulated Systems

**Figure 6:**
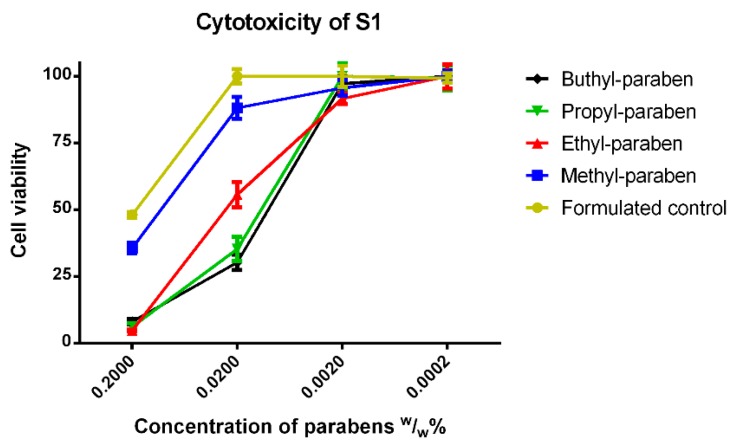
Cytotoxicity of the first formulated system (S1) consisting of 30% (*v*/*v*) glycerol and 0.002% (*v*/*v*) Polysorbate 20 measured by MTT-assay. Cell viability expressed as the percentage of the absorbance of the untreated control cells. Data expressed as mean ± SEM, *n* = 12. Cell viability of the samples at 0.2000; 0.0200; 0.0020; 0.0002 (*w*/*w*)% concentrations of different formulations containing parabens: formulated control: 48% ± 1.1%; 100% ± 2.7%; 100% ± 4%; 98% ± 1.8%; formulated MP: 36% ± 1.9%; 88% ± 4.1%; 96% ± 2.9%; 100% ± 2.1%; formulated EP: 5% ± 0.3%; 56% ± 2%; 92% ± 2%; 100% ± 4.6; formulated PP: 6% ± 1.3%; 35% ± 4.5%; 100% ± 4.9%; 99% ± 4.6%; formulated BP: 8% ± 1.1%; 30% ± 2.9%; 97% ± 2.6%; 100% ± 2.3%.

The cytotoxicity of S1 can be seen in Figure 6. The formulated control was highly toxic, and the cell viability was less than 50% compared to the untreated control at the original concentration. MP had the highest survivability from all esters, the second was EP. The results of the two longer parabens were not significantly different from each other at the tested concentrations. Moreover, the methyl paraben was not significantly different from the formulated control, but at the original concentration and at tenfold dilution, along with ethyl paraben, these derivatives were different from the longer ones. All statistical differences between the test solutions diminished at hundredfold and thousand-fold dilutions.

**Figure 7:**
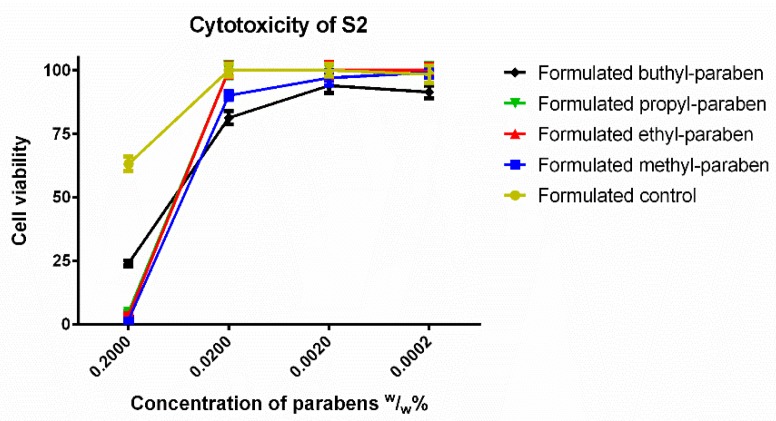
Cytotoxicity of the second formulated system (S2) consisting of 0.5% (*v*/*v*) Capryol PGMC™ and ethanol measured by MTT-assay. Cell viability expressed as the percentage of the absorbance of the untreated control cells. Data expressed as mean ± SEM, *n* = 12. Cell viability of the samples at 0.2000; 0.0200; 0.0020; 0.0002 (*w*/*w*)% concentrations of different formulations containing parabens: Formulated control: 63% ± 2.8%; 100% ± 2.5%; 100% ± 2.5%; 98% ± 3.4%; formulated MP: 2% ± 0.2%; 90% ± 1.8%; 97% ± 3.1%; 99% ± 2%; formulated EP: 4% ± 0.7%; 100% ± 2.7%; 100% ± 3.1%; 100% ± 2.5%; formulated PP: 5% ± 0.6%; 100% ± 3.2%; 100% ± 2.7%; 100% ± 2.7%; formulated BP: 24% ± 1.2%; 81% ± 2.6%; 94% ± 2.9%; 91% ± 2.5%.

In the case of S2 (Figure 7), BP had the highest cell viability at the original concentration, while the other parabens caused nearly total cell death. The tenfold dilution showed another ranking: propyl and ethyl paraben matched the results of the formulated control, while BP had slightly worse cell viability and MP was also significantly more toxic than the formulated control. After further dilution, all esters, except BP caused 100% cell viability. 

### 2.2. Antimicrobial Tests

#### 2.2.1. Antifungal Tests

In order to test the antimicrobial properties of parabens, three different concentrations were used. Cell viability was expressed as a percent of the absorbance of the positive control in the case of every species, respectively. The critical 50% cell viability threshold was presented with a line in each figure. Above this value a certain compound is considered ineffective in the case of antimicrobial activity, while below this line it has an inhibitory effect. We also formulated a control solution for every paraben to control their normal antimicrobial effects, without any additives. 

**Figure 8:**
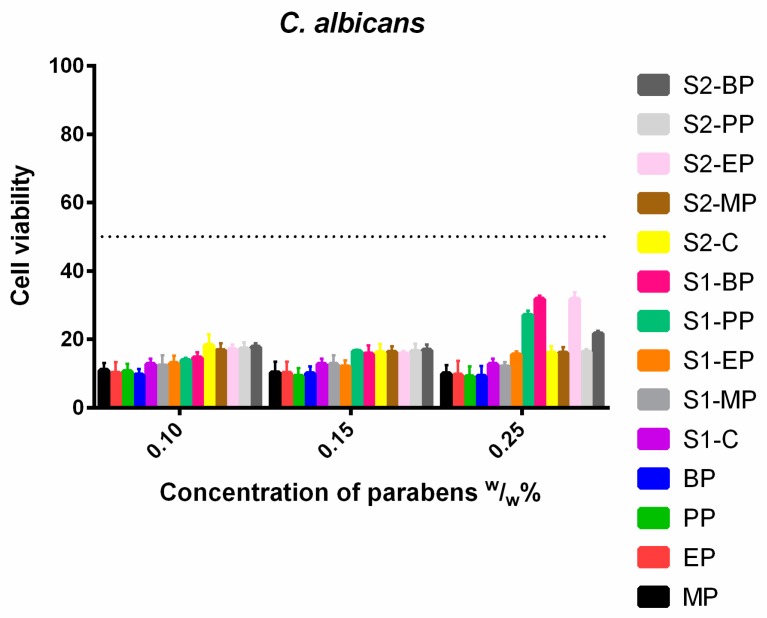
Cell viability of *C. albicans* against the control paraben solutions (MP; EP; PP; BP); the first system (S1-MP; S1-EP; S1-PP; S1-BP) and the second system (S2-MP; S2-EP; S2-PP; S2-BP). Cell viability expressed as the percentage of the absorbance of the untreated control. Data expressed as mean ± SEM, *n* = 4. Cell viability of fungal cells at 0.1; 0.15; 0.25 (*w*/*w*)% concentrations of different formulations containing parabens: MP: 11% ± 2.2%; 10% ± 3.2%; 10% ± 2.5%; EP: 10% ± 3.3%; 10% ± 3.4%; 10% ± 4.1%; PP: 11% ± 2.2%; 9% ± 2.3%; 9% ± 3%; BP: 10% ± 1.7%; 10% ± 2.2%; 9% ± 3%; formulated control of S1: 13% ± 1.7%; formulated MP of S1: 12% ± 3.1%; 13% ± 2.7%; 12% ± 1.4%; formulated EP of S1: 12% ± 2.3%; 12% ± 1.9%; 16% ± 0.9%; formulated PP of S1: 14% ± 0.7%; 16% ± 0.5%; 27% ± 1.3%; formulated BP of S1: 15% ± 1.6%; 16% ± 2.5%; 32% ± 1%; formulated control of S2: 18% ± 3.3%; 16% ± 2.5%; 16% ± 2%; formulated MP of S2: 17% ± 2.1%; 16% ± 1.7%; 16% ± 1.7%; formulated EP of S2: 17% ± 1.5%; 16% ± 0.8%; 32% ± 2%; formulated PP of S2: 17% ± 1.8%; 16% ± 2.2%; 16% ± 0.9%; formulated BP of S2: 18% ± 1.2%; 17% ± 1.7%; 22% ± 0.8%.

In the case of *C. albicans*, (Figure 8) there was no significant difference between formulated and non-formulated parabens, both the control solutions and the S1 and S2 solutions resulted in the same results. However, S1-PP, S1-BP and S2-EP had higher cell viability values than their controls, the formulations decreased the effectiveness of the parabens. There was no difference between the longer and the shorter esters.

**Figure 9:**
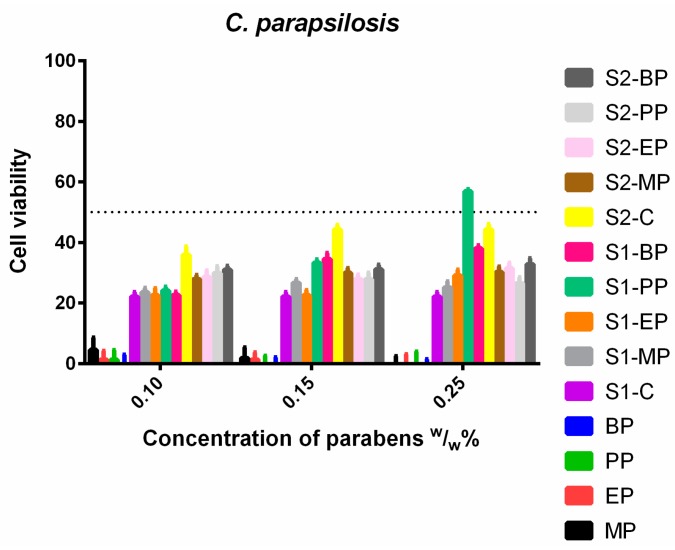
Cell viability of *C. parapsilosis* against the control paraben solutions (MP; EP; PP; BP); the first system (S1-MP; S1-EP; S1-PP; S1-BP) and the second system (S2-MP; S2-EP; S2-PP; S2-BP). Cell viability expressed as the percentage of the absorbance of the untreated control. Data expressed as mean ± SEM, *n* = 4. Cell viability of fungal cells at 0.1; 0.15; 0.25 (*w*/*w*)% concentrations of different formulations containing parabens: MP: 5% ± 4%; 2% ± 1.5%; 0% + 0.5%; EP: 1% ± 0.8%; 1% ± 0.3%; 0% + 0.1%; PP: 1% ± 0.1%; 0% + 0.4%; 0% + 0.2%; BP: 0% + 0.4%; 0% + 0.3%; 0% + 0.3%; formulated control of S1: 22% ± 1.5%; formulated MP of S1: 24% ± 1.4%; 27% ± 1.2%; 25% ± 2%; formulated EP of S1: 23% ± 2.3%; 23% ± 1.7%; 29% ± 1.9%; formulated PP of S1: 24% ± 1.3%; 33% ± 1.2%; 57% ± 0.7%; formulated BP of S1: 23% ± 1.1%; 34% ± 2%; 38% ± 0.9%; formulated control of S2: 36% ± 2.7%; 44% ± 1.5%; 44% ± 1.8%; formulated MP of S2: 28% ± 1.3%; 30% ± 1.6%; 30% ± 1.8%; formulated EP of S2: 29% ± 2.2%; 28% ± 1.5%; 31% ± 1.8%; formulated PP of S2: 30% ± 2.3%; 28% ± 2.1%; 27% ± 1.9%; formulated BP of S2: 31% ± 1.2%; 31% ± 1.5%; 33% ± 1.9%.

The investigation of *C. parapsilosis* (Figure 9) showed a high cell viability gap between the control parabens and the formulations. The control solutions totally eradicated the fungal cells, however, both formulations slightly increased their survivability. The increase of dissolved paraben did not reduce this gap, but even further weakened the antimicrobial effect of the parabens. At the highest concentration, the S1-PP no longer had inhibitory effect at all.

**Figure 10:**
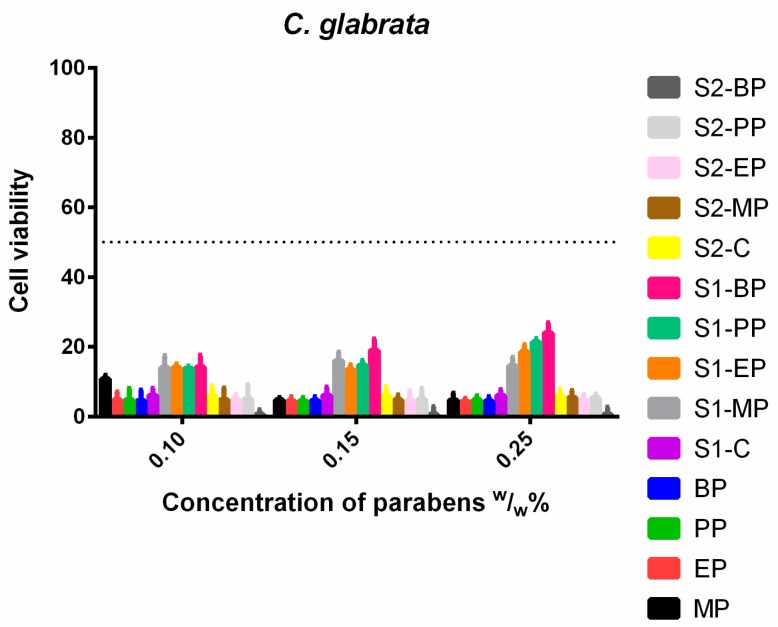
Cell viability of *C. glabrata* against the control paraben solutions (MP; EP; PP; BP); the first system (S1-MP; S1-EP; S1-PP; S1-BP) and the second system (S2-MP; S2-EP; S2-PP; S2-BP). Cell viability expressed as the percentage of the absorbance of the untreated control. Data expressed as mean ± SEM, *n* = 4. Cell viability of fungal cells at 0.1; 0.15; 0.25 (*w*/*w*)% concentrations of different formulations containing parabens: MP: 11% ± 1.1%; 5% ± 0.7%; 5% ± 2%; EP: 5% ± 2.3%; 4% ± 1.2%; 4% ± 0.9%; PP: 5% ± 3.2%; 4% ± 1%; 5% ± 1.2%; BP: 5% ± 3%; 5% ± 1.1%; 4% ± 1.2%; formulated control of S1: 6% ± 1.6%; formulated MP of S1: 14% ± 3.4%; 16% ± 2.5%; 15% ± 2.5%; formulated EP of S1: 14% ± 1.1%; 13% ± 1.4%; 19% ± 2%; formulated PP of S1: 14% ± 0.7%; 15% ± 1.4%; 21% ± 1%; formulated BP of S1: 14% ± 3.4%; 19% ± 3.2%; 24% ± 2.9%; formulated control of S2: 6% ± 2.9%; 6% ± 2.5%; 6% ± 2%; formulated MP of S2: 5% ± 3.4%; 5% ± 1.4%; 5% ± 2%; formulated EP of S2: 5% ± 1.4%; 5% ± 2.8%; 5% ± 1.7%; formulated PP of S2: 5% ± 4.1%; 5% ± 3.2%; 5% ± 1%; formulated BP of S2: 1% ± 0.9%; 1% ± 0.8%; 1% ± 0.9%.

*C. glabrata* was also sensitive to both the control and the formulated solutions (Figure 10), but the results of S1 were worse than S2 or the control. This lack of effectiveness increased with the growing concentration.

#### 2.2.2. Antibacterial Tests

**Figure 11:**
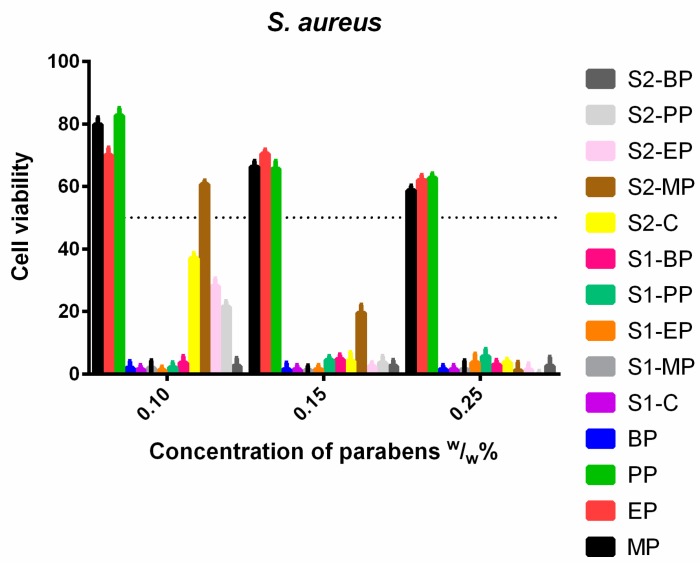
Cell viability of *S. aureus* against the control paraben solutions (MP; EP; PP; BP); the first system (S1-MP; S1-EP; S1-PP; S1-BP) and the second system (S2-MP; S2-EP; S2-PP; S2-BP). Cell viability expressed as the percentage of the absorbance of the untreated control. Data expressed as mean ± SEM, *n* = 4. Cell viability of bacterial cells at 0.1; 0.15; 0.25 (*w*/*w*)% concentrations of different formulations containing parabens: MP: 80% ± 2.2%; 66% ± 1.9%; 59% ± 1.5%; EP: 70% ± 2.4%; 70% ± 1.3%; 62% ± 1.6%; PP: 83% ± 2.3%; 66% ± 2.5%; 63% ± 1.4%; BP: 2% ± 1.1%; 1% ± 0.9%; 1% ± 0.4%; formulated control of S1: 1% ± 0.3%; formulated MP of S1: 2% ± 1.4%; 1% ± 0.5%; 1% ± 0.8%; formulated EP of S1: 1% ± 0.2%; 1% ± 0.4%; 4% ± 2.8%; formulated PP of S1: 2% ± 1.6%; 4% ± 1.3%; 6% ± 2.4%; formulated BP of S1: 4% ± 2.1%; 5% ± 1.6%; 3% ± 1.5%; formulated control of S2: 37% ± 1.6%; 4% ± 2.7%; 4% ± 1.1%; formulated MP of S2: 61% ± 1.2%; 20% ± 2.6%; 1% ± 0.5%; formulated EP of S2: 28% ± 2.4%; 2% ± 1.3%; 1% ± 0.9%; formulated PP of S2: 22% ± 1.7%; 4% ± 2.1%; 0% ± 0% +0.3%; formulated BP of S2: 3% ± 2.5%; 3% ± 1.8%; 3% ± 2.9%.

*S. aureus* (Figure 11) was not sensitive to the control solutions, and increasing doses of MP, EP and PP did not decrease the cell viability. Meanwhile, the ethanolic solution of BP and S1 proved to be highly effective. S2 containing Capryol PGMC™ apart from ethanol was also effective, with the exception of the formulated MP, which only passed the 50% limit at 1.5 (*w*/*w*)%. 

**Figure 12:**
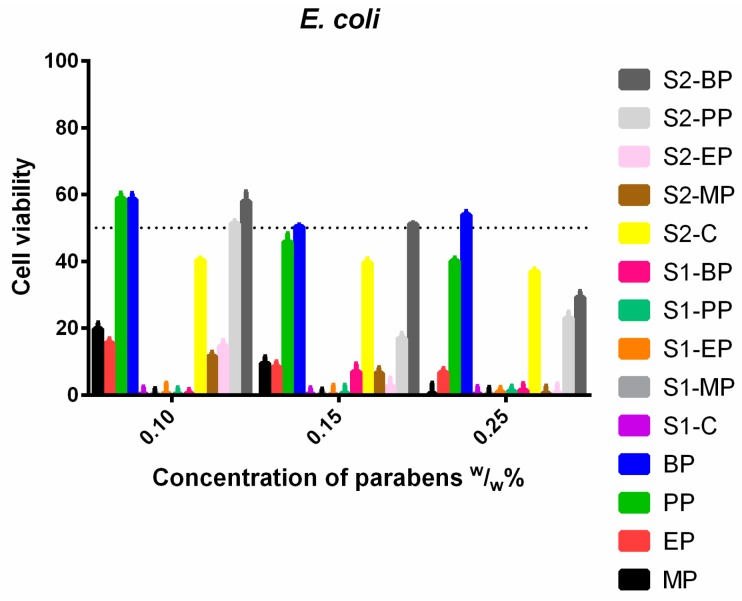
Cell viability of *E. coli* against the control paraben solutions (MP; EP; PP; BP); the first system (S1-MP; S1-EP; S1-PP; S1-BP) and the second system (S2-MP; S2-EP; S2-PP; S2-BP). Cell viability expressed as the percentage of the absorbance of the untreated control. Data expressed as mean ± SEM, *n* = 4. Cell viability of bacterial cells at 0.1; 0.15; 0.25 (*w*/*w*)% concentrations of different formulations containing parabens: MP: 20% ± 1.7%; 10% ± 2%; 0% + 0.1%; EP: 16% ± 1.2%; 8% ± 1.6%; 7% ± 1.2%; PP: 59% ± 1.6%; 46% ± 2.5%; 40% ± 0.9%; BP: 59% ± 1.8%; 50% ± 0.5%; 54% ± 1%; formulated control of S1: 0% + 0.4%; formulated MP of S1: 0% ± 0.3%; 0% ± 0.1%; 0% ± 0.2%; formulated EP of S1: 0% + 0.1%; 0% + 0.2%; 1% ± 0.4%; formulated PP of S1: 0% + 0.2%; 0% + 0.4%; 1% ± 0.4%; formulated BP of S1: 0% + 0.3%; 7% ± 2.4%; 1% ± 1%; formulated control of S2: 40% ± 0.6%; 40% ± 1.1%; 37% ± 0.9%; formulated MP of S2: 12% ± 1.2%; 7% ± 1.6%; 1% ±0.2%; formulated EP of S2: 15% ± 1.7%; 3% ± 2.4%; 1% ± 0.7%; formulated PP of S2: 51% ± 0.8%; 17% ± 1.5%; 23% ± 1.9%; formulated BP of S2: 58% ± 2.9%; 51% ± 0.4%; 29% ± 1.8%.

*E. coli* (Figure 12) had resistance against EP and BP, except for the S1, which was very effective against it. The addition of a surface-active agent in S2 could increase the antimicrobial properties of BP and PP as they showed greater inhibitory effect than the normal ethanolic solutions. S1 showed higher efficacy than the other solutions.

**Figure 13:**
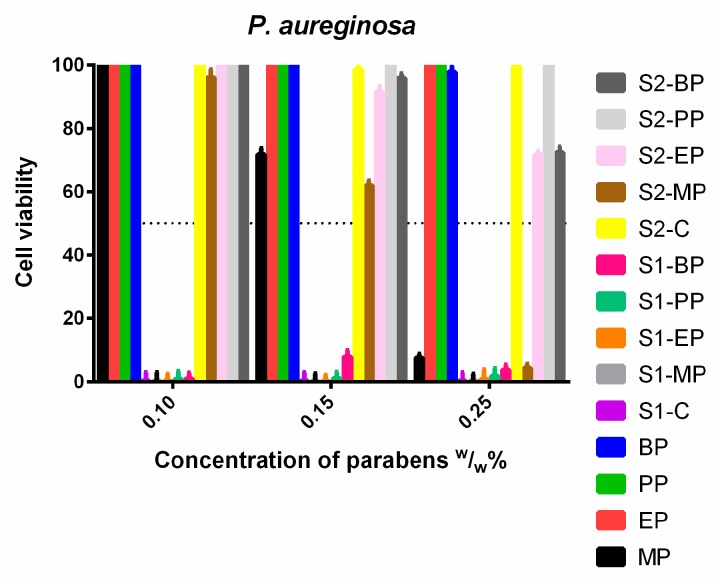
Cell viability of *P. aeruginosa* against the control paraben solutions (MP; EP; PP; BP); the first system (S1-MP; S1-EP; S1-PP; S1-BP) and the second system (S2-MP; S2-EP; S2-PP; S2-BP). Cell viability expressed as the percentage of the absorbance of the untreated control. Data expressed as mean ± SEM, *n* = 4. Cell viability of fungal cells at 0.1; 0.15; 0.25 (*w*/*w*)% concentrations of different formulations containing parabens: MP: 100% ± 2.2%; 72% ± 2.0%; 7% ± 1.2%; EP: 100% ± 2.1%; 100% ± 2.3%; 100% ± 1.7%; PP: 100% ± 1.7%; 100% ± 1.2%; 100% ± 1.8%; BP: 100% ± 1.3%; 100% ± 1.4%; 98% ± 2%; formulated control of S1: 0% + 0.4%; formulated MP of S1: 0% + 0.2%; 0% + 0.3%; 0% + 0.1%; formulated EP of S1: 0% + 0.2%; 0% + 0.2%; 0% + 0.3%; formulated PP of S1: 1% + 0.5%; 1% + 0.3%; 1.8% + 0.4%; formulated BP of S1: 1% + 0.1%; 8% ± 2.1%; 4% ± 1.7%; formulated control of S2: 100% ± 2.6%; 99% ± 1.4%; 99% ± 1.7%; formulated MP of S2: 96% ± 2.5%; 62% ± 1.5%; 4% ± 1.3%; formulated EP of S2: 100% ± 2%; 92% ± 1.7%; 72% ± 1.3%; formulated PP of S2: 100% ± 2.1%; 100% ± 1.6%; 100% ± 1.8%; Formulated BP of S2: 100% ± 2%; 96% ± 1.4%; 72% ± 1.9%.

*P. aeruginosa* (Figure 13) showed the widest spectrum of resistance. The ethanolic EP, PP and BP could not inhibit its growth at all, like PP and BP in S2. The presence of Capryol PGMC™ was also advantageous for the EP and BP, their effectiveness was highly increased, but they still could not reach a 50% inhibitory rate. All derivatives formulated in S1 were totally effective in every concentration, and methyl paraben was also effective at the highest dose in S2 and the control ethanolic solutions. 

## 3. Discussion

The microbial stability of any oral pharmaceutical product until its expiry date is essential regardless if the product was contaminated during its application. However, the use of preservatives is a cheap way to protect any product, there are authorized drugs on the market with ineffective microbial protection [28]. In our study, we formulated two different co-solvent systems:

S1 which contained 30% (*v*/*v*) glycerol and 0.002% (*v*/*v*) Polysorbate 20 (HLB value: 16.7) and S2 which contained 0.5% (*v*/*v*) Capryol PGMC™ (HLB value: 5) and parabens in the form of their 70% (*v*/*v*) ethanolic solutions [25].

The basis of selection was to use one co-solvent, different surfactants with high and moderate HLB values and preservatives (parabens) in our investigations, because these excipients are officially widely applied in liquid, oral pharmaceutical formulations. In order to comply with EMEA guidelines, these authorized excipients were used in safe concentrations [29]. The cytotoxicity of ethanol and glycerin as co-solvents were also tested on Caco-2 cell line and their cytocompatible concentrations were determined. The safe 0.5 (*v*/*v*)% ethanol concentration was controlled in OTC products for children [30]. We applied ethanol as co-solvent in 1.75 (*v*/*v*)%, 1.4 (*v*/*v*)%, 0.14 (*v*/*v*)%, 0.014 (*v*/*v*)% concentration range and these concentrations proved to be cytocompatible on Caco-2 cells. Ethanol can increase the solubility of several drugs, such as COX-2 inhibitors even at low concentration and it showed cytotoxic properties at 10% (*v*/*v*) on Caco-2 cells [31,32]. 

The effective glycerol concentration for enhancing the solubility of different active pharmaceutical ingredients (APIs) was proved from 20% (*v*/*v*) [33]. The concentration of glycerol of our complex systems was used from 30 (*v*/*v*)%, 3 (*v*/*v*)%, 0.3 (*v*/*v*)%, to 0.03 (*v*/*v*)% which were lower than the inhibitory concentration.

The selection of Polysorbate 20 and Capryol PGMC based on our previous experiments [25]. It was found that the safe concentration range of Polysorbate 20 was 0.002 (*v*/*v*)%, 0.0002 (*v*/*v*)%, 0.00002 (*v*/*v*)% and 0.000002 (*v*/*v*)%. The HLB value of Polysorbate 20 is high and effective solubilizing properties can be presented. Surfactant with lower HLB value was Capryol PGMC and cytocompatible concentration range (0.5 (*v*/*v*)%, 0.05 (*v*/*v*)%, 0.005 (*v*/*v*)%, 0.0005 (*v*/*v*)%) was confirmed in our study.

Based on our experimental design, we tested two complex formulations containing different parabens to evaluate their cytotoxic and antimicrobial interference and to investigate how these materials influence the safety and efficacy of parabens on human cells, bacteria and fungi—*E. coli*, *P. aeruginosa*, *S. aureus*, *C. albicans*, *C. parapsilosis*, *C. glabrata*. The antimicrobial stability can depend on the formulation and the different excipients used in the product. Ribes et al. have found that the antimicrobial effect of cinnamon leaf essential oil can be heavily enhanced by a nanoemulsion of emulsifying agents [34]. Han et al. also investigated the effects of preservatives and they found that there are multiple preservatives which influence the stability of Diprivan^®^ injection (an intravenous anaesthetic emulsion) in an undesirable way [35]. 

As it was found, S1 and S2 complex systems had significant concentration dependent cytotoxic effect on Caco-2 cells. In the in vitro Caco-2 cell line investigations, our systems were diluted with phosphate buffered saline (PBS). We prepared our solutions 10 minutes before the starting point of the incubations of Caco-2 cells to prevent any accelerated hydrolysis caused by the other excipients as the transesterification of parabens by Caco-2 cells after long incubation times was reported [36], the incubation time was minimized in our MTT-test in order to avoid any significant conversion. Our experiment was supported by the results of Tomankova et al., because they showed that parabens were chemically stable, and they only converted by spontaneous hydrolysis in aqueous solutions after months of storage [37]. 

It was found that in our test systems the relative toxicity of parabens was different in these two formulated systems, and the ranking of toxicity was not general. The parabens alone, presented the ranking of BP > PP > EP > MP as butyl paraben was the most toxic. In most scientific studies, such as the publication of Dagher et al., it was reported that the cytotoxicity increased with the length of alkyl chains as well [38]. They investigated that MP was the least toxic on MCF7 breast cancer cells, while the cytotoxicity of more lipophilic parabens (BP and benzyl paraben) eventually increased. While the S1 had identical results, in S2, the other excipients could modify the ranking, BP at the highest concentration was the safest paraben.

In S1, in the presence of glycerol and Polysorbate 20, MP was the least toxic compound, while the other parabens with longer alkyl chains had similar curves and reached nearly total cell death at 0.2 (*w*/*w*)%. At tenfold dilution, the cell viability value of MP was significantly higher than the other parabens, but at that concentration, EP became distinguishable from the more lipophilic derivatives. The more diluted solutions showed no toxicity at all. In S1, the low cell viability values can be explained by the increased osmotic pressure of glycerol which has already been proved on human cells [39]. The Polysorbate 20, which was found to have an IC_50_ value of 0.004% (*v*/*v*) can easily distort the cell membrane [25]. On A549 human lung cancer cells and HUVECs even a very minimal concentration of Polysorbate 20 caused nearly total cell death [40]. Polysorbate is known to increase the transport of certain drug molecules through cell membranes by a membrane component solubilization mechanism [41]. It was supposed that this dual effect—the osmotic stress and the distortion of the cell membrane—was responsible for the dramatically low cell viability of Caco-2 cells. This may well mean that when a pharmaceutical product has a high osmotic pressure or contains a surfactant with high HLB value, the cytotoxicity must be reconsidered, because these excipients can enhance the toxicity of each other in a synergetic way.

In S2, in the presence of ethanol and Capryol PGMC™, the results were controversial. At the original concentration, BP was the least toxic compound, but at tenfold dilution, it was not statistically distinguishable from MP, while these two were significantly different from both the control, and the other esters. The formulated control of the second system had lower cell viability than, the control of the first system. The parabens alone still showed the same ranking as the S1, it can be assumed, that the surfactant with low HLB value were responsible for the lower toxicity of BP. 

In the case of the antimicrobial tests, our systems were tested on clinically relevant pathogens which can represent a variety of possible contaminating agents. However, the exact mechanism of action of parabens is unknown, and there are various papers describing different pathways. Bredin et al. found that on *E. coli*, PP had similar potassium efflux-creating effects as polymyxin. The site of action was the porin channels, as their specific blockers could protect the bacteria from damage [42]. It was also reported that they could distort the structure of the cell membrane [43]. It was also a menacing result, as parabens showed higher affinity to distort phospholipids in mammalian cell membranes than bacterial ones. A general theory of action, is that like other weak acids used as preservatives (sorbates, benzoates, propionates, etc.) 4-hydroxybenzoic acid at acidic pH penetrates the cells in a nonionized way and in the cytosol, it dissolves and the resulting H^+^ act as the main metabolic disruptive force [44]. According to Thong et al., MP had no direct dependence on the pH, for *A. sulphureus* and *P. viridicatum*, nevertheless, it was able to drastically decrease the production of ochratoxin A for both species [45]. Er et al. reported similar results, where the difference between the inhibition on the growth and the biofilm formation at pH 6 and pH 7 on *Salmonella* strains was not significant [46]. A possible way of resistance to parabens can be the hydrolysis of esters by enzymatic activity [47]. 

As for the antibacterial results, it can be said that inhibitory potential of parabens differed greatly. While *E. coli*, a Gram-negative bacterium showed resistance towards PP and BP, another Gram-negative strain, *P. aureginosa*, had nearly total resistance towards all parabens, and only the highest dose of MP could effectively inhibit it. The S1 formulation was very effective against both species, even the formulated control, and without any additional paraben could eradicate the microbes. This is greatly advantageous, as the resistance of *P. aeruginosa* towards many disinfectants in known [48]. Formulation S2 which only contained an additional surface-active agent, could not drastically increase the effect of parabens, and only a small decrease of cell viability could be measured. The formulated control of S2 had no effect on *P. aeruginosa*, but was effective against *E. coli*, as this bacterium was overall more sensitive to the parabens. The only Gram-positive bacterium, *S. aureus,* was resistant to MP, EP and PP and susceptible only to BP. S1 caused total cell death and the presence of Capryol PGMC™ could effectively increase the effect of the shorter esters. It can be stated that Gram-positive and negative strains have different sensitivity towards surface-active agents and as the results indicate, the Gram-positive species are more vulnerable to them, supposedly due to their different cell wall structure. Smaoui et al., found that 1% (*w*/*w*) MP had limited effect on *S. aureus*, which correlates with our results [49]. The controversial susceptibility to the different parabens means, that for the tested bacteria, there was no linear relationship between the length of alkyl chain and the antimicrobial effect as it was stated in literature [50], but rather every ester must be tested against a given microbe in a solution that has the composition of the final pharmaceutical product. 

The cell wall of *Candida* species is different from its bacterial counterparts, it is not a rigid structure, but a more flexible outer layer of the cells, capable of structural changes [51]. The cell wall of *C. albicans* and *C. glabrata* are relatively well described. They are similar, the main reported difference being their adhesin-like proteins, which are mostly important for the pathogenicity and mask them from the immune system [52]. However, the cell wall of *C. parapsilosis* is not described precisely, but it also has similar attributes that of the *C. albicans* [53]. Despite this, *C. parapsilosis* was the most resistant of all the tested yeasts, and the only one where the 50% cell viability threshold was reached, in case of the highest concentration of PP formulated in the first system. *C. parapsilosis* was also sensitive to ethanolic solutions of parabens, but both S1 and S2 had less effect on it as cell viability was always above 20% for the formulated systems. For *C. glabrata* and *C. albicans*, there was a small, but persistent gap between the formulated and the non-formulated test solutions. At the same time, it could be noted, that in most scenarios MP was as effective as the longer parabens which correlates well with the broad range study of Matos et al. [54]. On all fungi, but mostly on *C. parapsilosis*, a very interesting pattern could be seen: the control samples, which only consists of the given ester, ethanol and PBS had better inhibition effect than the formulated systems. Nor a high amount of glycerol—and the osmotic pressure of it—nor the two surfactant, could enhance the effect of parabens, on the contrary, they increased the survivability of the fungi. The exact mechanism is poorly understood and needs further investigation. Also, for the tested fungi, no linear relationship was found between the length of alkyl chain and the antimicrobial activity, as stated in previous results [55].

Generally, the S1 totally eradicated all bacteria, so they were less resistant to the dual effect of osmotic pressure and solubilization, while S2 had limited effects on them. The fungi were mostly sensitive to the parabens, but both formulations proved to be less effective on them, than the ethanolic solutions of parabens. The two systems presented similar patterns of cytotoxicity on human cells, but as it was seen on the bacteria and the fungi, but the length of the paraben alone is not informative to to predict the cytotoxic and antimicrobial properties. 

## 4. Materials and Methods

### 4.1. Materials

Methyl 4-hydroxybenzoate and glycerol were purchased from Hungaropharma (Budapest, Hungary). Ethyl 4-hydroxybenzoate was obtained from Acros Organics (Geel, Belgium), propyl 4-hydroxybenzoate from Alfa Aesar (Karlsruhe, Germany), butyl 4-hydroxybenzoate from TCI (Zwijndrecht, Belgium). Capryol PGMC™ was a kind gift from Gattefossé (Lyon, France). The 3-(4,5-dimethylthiazol-2-yl)-2,5-diphenyltetrazolium bromide (MTT), Dulbecco’s Modified Eagle’s Medium (DMEM), phosphate buffered saline (PBS), Trypsin-EDTA, Heat-inactivated fetal bovine serum (FBS), L-glutamine, non-essential amino acids solution, gentamycin, RPMI-1640 broth with L-glutamine and Mueller-Hinton broth were purchased from Sigma-Aldrich (Budapest, Hungary). Non-essential amino acids solution and penicillin-streptomycin mix were obtained from VWR (Debrecen, Hungary), L-glutamine and GlutaMax™ supplement was from Thermo Fisher (Budapest, Hungary).

### 4.2. Cell Culture

Caco-2 cell line was obtained from the European Collection of Cell Cultures (ECACC, Salisbury, United Kingdom). Cells were grown in plastic cell culture flasks in Dulbecco’s Modified Eagle’s Medium, supplemented with 3.7 g/L NaHCO_3_, 10% (*v*/*v*) heat-inactivated fetal bovine serum (FBS), 1% (*v*/*v*) non-essential amino acids solution, 1% (*v*/*v*) L-glutamine, 100 IU/mL penicillin, and 100 µg/mL streptomycin at 37 °C in an atmosphere of 5% CO_2_. The cells were routinely maintained by regular passaging and glutamine was supplemented by GlutaMax™. The cells used for cytotoxic experiments were between passage numbers 20 and 40.

### 4.3. Cytotoxicity Tests

The cytotoxic effects of the various solutions were evaluated using the 3-(4,5-dimethylthiazol-2-yl))-2,5-diphenyltetrazolium bromide (MTT) cytotoxicity method. Caco-2 cells in complete medium were seeded on 96-well plate at a final density of 10.000 cells/well. After 7 days, the medium was removed, and the cells were incubated for 30 min with the test solutions. All test solutions were prepared 10 min before the start of incubation. The samples were removed, and a 5 mg/mL MTT solution (MTT salt solved in PBS) was added to each well. The plates were incubated for 3 h, then the MTT solution was removed and 0.1 mL of a solution of isopropanol—1 M hydrochloride acid (25:1) was added to dissolve the formed formazan crystals. The absorbance was measured at 570 nm against a 690 nm reference with a Thermo-Fisher Multiskan Go (Thermo Fisher, Budapest, Hungary) microplate reader. Cell viability was expressed as a percent of the cell viability of the untreated control cells, which were incubated with PBS for 30 min.

### 4.4. Antimicrobial Tests

Antibacterial and antifungal susceptibility testing was performed using standard broth microdilution method in accordance with the recent EUCAST protocols (E.Dis 5.1, E.Def 7.3.1) against *Pseudomonas aeruginosa* (ATCC^®^ 27853™), *Escherichia coli* (ATCC^®^ 25922™), *Staphylococcus aureus* (ATCC^®^ 43300™), *Candida albicans* (ATCC^®^ 10231™), *C. parapsilosis* (ATCC^®^ 22019™) and *C. glabrata* (ATCC^®^ 90030™) [56,57]. Briefly, the broth microdilution assays were performed using 96-well standard microtitre plates, where the given concentrations of the tested compounds are prepared in RPMI-1640 and Mueller-Hinton medium for fungal species and bacteria, respectively. All test solutions were prepared 10 min before the start of incubation. The final volume of each well contained 100 µL from the tested compounds and 100 µL fungal or bacterial inoculum. The inoculum size was 2 × 10^3^ cells/mL and 5 × 10^5^ CFU/mL for Candida species and bacteria species, respectively. Plates were incubated for 24 h at 37 °C. After the incubation period, the absorbance was measured by a Thermo-Fisher Multiskan Go (Thermo Fisher) microplate reader at 492 nm and 600 nm for fungal species and bacterial species, respectively. Prominent inhibition was determined based on turbidity as at least 50% growth reduction compared with the compound-free control. Percent change in turbidity was calculated on the basis of absorbance (A) as 100% × (A_well_ − A_background_)/(A_compound-free well_ − A_background_). The background was measured from the microbe-free well.

### 4.5. Statistical Analysis

All data were analysed using GraphPad Prism (version 6; GraphPad Software, Inc., La Jolla, CA, USA). In case of MTT-assay results, the data was presented as means ± SEM. Each cell viability value represents the mean of twelve independent, parallel wells, with the highest and lowest absorbance values were excluded when calculating the mean. After that, at each concentration, the means of different solutions where compared with one-way ANOVA test followed by Tukey’s test when all solutions were compared to each other (Table 3).

Previously, all data group were analysed with Shapiro-Wilk test for Gaussian distribution and Bartlett’s test for equal variances. In each case we used significance level *p* < 0.05. In case of antimicrobial tests the results of four parallel, independent wells were represented as means ± SEM. 

## 5. Conclusions

Different test systems containing parabens as preservatives were formulated (S1: glycerol and polysorbate 20; S2: ethanol and Capryol PGMC™) to observe any kind of correlation between the cytotoxicity and microbial inhibitory potential of parabens. The cytocompatibility and antimicrobial activity of parabens depends on the length of alkyl chains, the chemical environment and the targeted cells. The IC_50_ values of different parabens can be modified by other excipients. Co-solvents (glycerol, ethanol) and surfactants (Polysorbate 20 and Capryol PGMC) can modify the cytotoxicity and antimicrobial activity of different parabens. The mechanism of these connections may be different, because surfactants may solubilize membrane components and disrupted the membrane integrity, while co-solvents resulted in the damage of mitochondria in the cells. It can be concluded that in vitro data are not necessarily predictive, but complemented with in vivo experiments could be informative. 

## Figures and Tables

**Table 1:** Composition of test solutions for cytotoxicity tests.

Component	S1	S2
**Paraben**	0.2 *w*/*w*%, 0.02 *w*/*w*%, 0.002 *w*/*w*%, 0.0002 *w*/*w*%	
**Glycerol**	30 *v*/*v*%, 3 *v*/*v*%, 0.3 *v*/*v*%, 0.03 *v*/*v*%	-
**Polysorbate 20**	0.002 *v*/*v*%, 0.0002 *v*/*v*%, 0.00002 *v*/*v*%, 0.000002 *v*/*v*%	-
**Capryol PGMC™**	-	0.5 *v*/*v*%, 0.05 *v*/*v*%, 0.005 *v*/*v*%, 0.0005 *v*/*v*%
**Ethanol**	-	1.4 *v*/*v*%, 0.14 *v*/*v*%, 0.014 *v*/*v*%, 0.0014 *v*/*v*%
**PBS**	solvent, used for tenfold, hundredfold, thousand-fold dilution	

**Table 2:** Composition of test solutions for antimicrobial tests.

Component	S1	S2	Control
**Paraben**	0.1 *w*/*w*%, 0.15 *w*/*w*%, 0.25 *w*/*w*%		
**Glycerol**	30 *v*/*v*%	-	-
**Polysorbate 20**	0.002 *v*/*v*%	-	-
**Capryol PGMC™**	-	0.5 *v*/*v*%	-
**Ethanol**	-	0.7 *v*/*v*%, 1.05 *v*/*v*%, 1.75 *v*/*v*%	0.7 *v*/*v*%, 1.05 *v*/*v*%, 1.75 *v*/*v*%
**RPMI-1640**	solvent for antifungal tests		
**Mueller-Hinton broth**	solvent for antibacterial tests		

**Table 3:** Result of Tukey's multiple comparison test performed on the results of Caco-2 cells. FC: formulated control, Methyl: formulated methyl-paraben, Ethyl: formulated ethyl-paraben, Propyl: formulated propyl-paraben, Butyl: formulated butyl-paraben. * *p* < 0.05; ** *p* < 0.01; *** *p* < 0.001, **** *p* < 0.0001.

ANOVA Followed by Tukey’s Multiple Comparisons Test	Level of Significance
Parabens alone, 0.2% Methyl vs. Ethyl	ns
Parabens alone, 0.2% Methyl vs. Propyl	**
Parabens alone, 0.2% Methyl vs. Butyl	****
Parabens alone, 0.2% Ethyl vs. Propyl	**
Parabens alone, 0.2% Ethyl vs. Butyl	****
Parabens alone, 0.2% Propyl vs. Butyl	ns
Parabens alone, 0.02% Methyl vs. Ethyl	ns
Parabens alone, 0.02% Methyl vs. Propyl	**
Parabens alone, 0.02% Methyl vs. Butyl	*
Parabens alone, 0.02% Ethyl vs. Propyl	*
Parabens alone, 0.02% Ethyl vs. Butyl	ns
Parabens alone, 0.02% Propyl vs. butyl	ns
Parabens alone, lower concentrations	all are insignificant
S1, 0.2% Formulated control vs. Methyl	*
S1, 0.2% FC vs. Ethyl	****
S1, 0.2% FC vs. Propyl	****
S1, 0.2% FC vs. Butyl	****
S1, 0.2% Methyl vs. Ethyl	****
S1, 0.2% Methyl vs. Propyl	****
S1, 0.2% Methyl vs. Butyl	****
S1, 0.2% Ethyl vs. Propyl	ns
S1, 0.2% Ethyl vs. Butyl	ns
S1, 0.2% Propyl vs. Butyl	ns
S1, 0.02% FC vs. Methyl	*
S1, 0.02% FC vs. Ethyl	****
S1, 0.02% FC vs. Propyl	****
S1, 0.02% FC vs. Butyl	****
S1, 0.02% Methyl vs. Ethyl	****
S1, 0.02% Methyl vs. Propyl	****
S1, 0.02% Methyl vs. Butyl	****
S1, 0.02% Ethyl vs. Propyl	***
S1, 0.02% Ethyl vs. Butyl	***
S1, 0.02% Propyl vs. Butyl	ns
S1 lower concentrations	all are insignificant
S2, 0.2% Formulated control vs. Methyl	****
S2, 0.2% FC vs. Ethyl	****
S2, 0.2% FC vs. Propyl	****
S2, 0.2% FC vs. Butyl	****
S2, 0.2% Methyl vs. Ethyl	ns
S2, 0.2% Methyl vs. Propyl	ns
S2, 0.2% Methyl vs. Butyl	**
S2, 0.2% Ethyl vs. Propyl	ns
S2, 0.2% Ethyl vs. Butyl	**
S2, 0.2% Propyl vs. Butyl	**
S2, 0.02% FC vs. Methyl	*
S2, 0.02% FC vs. Ethyl	ns
S2, 0.02% FC vs. Propyl	ns
S2, 0.02% FC vs. Butyl	**
S2, 0.02% Methyl vs. Ethyl	*
S2, 0.02% Methyl vs. Propyl	*
S2, 0.02% Methyl vs. Butyl	ns
S2, 0.02% Ethyl vs. Propyl	ns
S2, 0.02% Ethyl vs. Butyl	*
S2, 0.02% Propyl vs. Butyl	*
S2 lower concentrations	all are insignificant
